# Nonspecific Symptoms in a Rare Case of Urethral Adenocarcinoma in a 58-Year-Old Female

**DOI:** 10.1155/2018/9010246

**Published:** 2018-05-23

**Authors:** Line Winther Gustafson, Anne Gamst Christiansen, Huda Majeed, Peter Humaidan

**Affiliations:** ^1^Department of Gynaecology and Obstetrics, Region Hospital Viborg and Skive, Heiberg Allé 4, 8800 Viborg, Denmark; ^2^The Fertility Clinic, Region Hospital Viborg and Faculty of Health, Aarhus University, Aarhus, Denmark

## Abstract

Cancer of the urethra is very rare with an age-adjusted incidence of only 0.6 per million women in Europe. The etiology is multifactorial and the incidence increases with age, with the highest rates in patients 75 years or older. We herein describe a 58-year-old woman referred to our unit due to pollakisuria and repeated lower urinary tract infections. The gynecological examination revealed a suspect area in the anterior wall of vagina. Subsequently, ultrasound examination, MRI, and PET-CT scan followed by vaginal biopsies revealed a urethral adenocarcinoma.

## 1. Introduction

Urethral cancer is very rare. The annual age-adjusted incidence rate for women is 1.5 per million in the United States and only 0.6 per million women across Europe [1, 2]. The 5-year relative survival rate for cancer of the urethra is 54%. The predominant type is the transitional cell carcinoma constituting 64% of the cases while the squamous cell carcinoma and adenocarcinoma account for 16% and 10%, respectively [1]. There is a peak incidence in the age group of 80–85 years [3].

The ethology is multifactorial. Thus, recurrent urinary tract infections, sexual-transmitted diseases, and urethral stricture seem to increase the risk of urethral carcinoma [1]. Also, Human Papilloma Virus (HPV) plays a role in urethral cancer as shown by Wiener and Walther who reported the presence of HPV-16 and HPV-18 in 59% of female patients with urethral carcinoma [4].

We herein present a rare case of a 58-year-old woman with urethral adenocarcinoma.

## 2. Case Presentation

In November 2016, a 58 year-old woman was referred from her general practitioner to our Gynecological Department. Her main complaints were up to 16–18 micturitions a day and repeated bladder infections despite continuous antibiotic treatment with either Pivmecillinam (Selexid®) or Nitrofurantoin. She had a feeling that she could not empty her bladder.

Due to the organization of our outpatient clinic, a nurse with a special interest in incontinence initially saw the patient. At this visit an uroflowmetry showed obstruction, a voiding time of 78 sec, and residual urine of 190 ml.

Input and output tables showed daily intakes of 1700–1800 ml and a total urine production of 1310–1860 ml. At her first visit the patient was instructed to perform double or triple voiding, and further she was recommended local treatment with estrogens (Vagifem® or Estring®).

After one month, she was referred to the outpatient clinic to see an urogynecologist. She was in pain and was not able to empty her bladder properly through the night. Moreover, during the previous months, she had some abnormal bleeding from the vagina and blood-stained urine.

A gynecological examination was performed which revealed a palpable solid mass located in the anterior wall of the vagina.

The anterior and posterior vaginal wall presented without prolapse. Uterus was well suspended and the cervix appeared normal. After examination, a small area in the anterior wall of the vagina was seen to bleed.

Transvaginal ultrasound showed a bladder containing a total of 250 ml urine, a normal uterus with a thin endometrium of 1,4 mm, and two normal adnexa.

The solid area was located in close contact with the anterior wall of the vagina and the urethra was measured to be 29 × 28,8 mm on ultrasound.

Due to the urinary retention and pain, an attempt to apply a urinary catheter was done; however, this was not possible due to obstruction. Blood samples from the bleeding area in the anterior wall of the vagina and from the urethra were sent to the pathologist for further examination. An urologist was contacted and an ultrasound-guided suprapubic catheter was placed.

**Figure 1:**
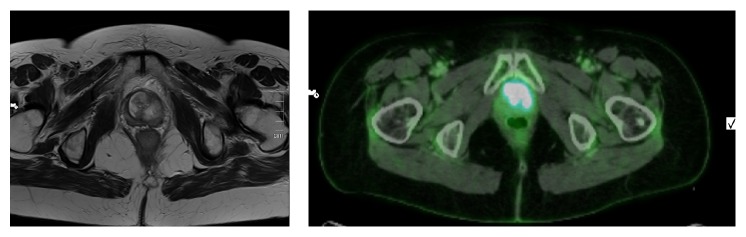
MRI and PET-CT scan visualizing the tumor.

The patient was further examined having a cystoscopy with biopsies, a MRI scan, and a PET-CT scan, revealing a urethral adenocarcinoma measuring 4 × 4,5 cm with suspected lymph node affection of the iliac vessels on the left side (Figure 1).

**Figure 2:**
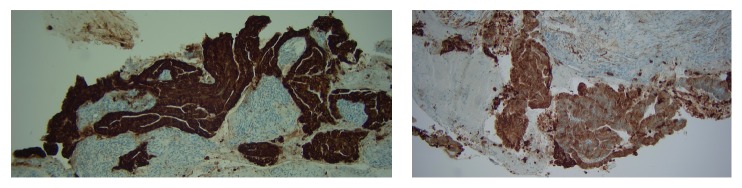
Histological pictures of the tumor positive for cytokeratin-20 and cytokeratin-7, respectively.

In January 2017, 19 lymph nodes were removed laparoscopically. All 19 lymph nodes were normal without any malignancy. The finale staging diagnosis was a large T3, M0, N0 tumor (Figure 2). Due to the size and close relation of the tumor to the pubic bone, it was not possible to gain adequate surgical margins if performing radical surgery. Therefore, together with the patient it was decided that she should receive local radiation of 50 Gy on 30 fractions and subsequently brachytherapy without concomitant chemotherapy with Cisplatin.

## 3. Discussion

Urethral cancer is very rare with an annual age-adjusted incidence rate for women being 1.5 per million in the United States and only 0.6 per million women across Europe [1, 2]. Patients often present with advanced clinical stages of the disease. The initial symptoms can be macroscopic hematuria or bloody urethral discharge. Lower abdominal pain, dyspareunia, and bladder outlet obstruction can also be seen due to the growing tumor mass [5].

MRI scan is used for local staging of the tumor, while imaging for regional lymph node metastases involves either MRI-CT or (PET-) CT scan. In women, the proximal 1/3 of urethral drains into the pelvic lymph nodes and the distal 2/3 drains into the superficial or deep inguinal lymph nodes. Finally, a CT scan is used for assess the possible presence of metastases in the abdomen and thorax [5]. In a study by Derksen et al. 46% of the patients were diagnosed as having an advanced form of the disease (tumor stage and TNM stages III or IV) [3].

Treatment consists of surgery, radiotherapy, chemotherapy, brachytherapy, and often a combination of these. Previously, Derksen et al. reported an overall survival rate of stages 0–II, stage III, and stage IV of 67, 53, and 17%, respectively. Moreover, Dalbagni et al. found that the 5-year overall survival was 32%, while Visser et al. reported the 5-year relative survival rate for cancer of the urethra to be 54% [1, 3, 6]. Surgery can be performed either as partial urethrectomy or as a radical urethrectomy depending on the size and location of the tumor [5]. In a study from DiMarco et al. (2004) including 53 patients, 26 patients underwent partial urethrectomy and 27 patients radical extirpation. Twenty patients had radiation therapy, mostly external beam radiotherapy, and only 2 patients received brachytherapy. Of the 26 patients who underwent partial urethrectomy for a T1-T3 tumor, 22% had urethral recurrence, which must be considered high [7]. In this case, the tumor size and the close location to the pubic bone made radical surgery impossible.

## 4. Conclusion

Urethral cancer can present with different nonspecific symptoms from the urinary tract. Awareness of urethral cancer is important in daily clinical practice as it may present also in women under the age of sixty.
